# Impact of *Helicobacter pylori* Infection upon the Evolution and Outcome of Pediatric Immune Thrombocytopenic Purpura: A Comprehensive Review

**DOI:** 10.3390/diagnostics13203205

**Published:** 2023-10-13

**Authors:** Maria Oana Săsăran, Cristina Oana Mărginean, Ana Maria Koller

**Affiliations:** 1 Department of Pediatrics 3, University of Medicine, Pharmacy, Sciences and Technology George Emil Palade from Târgu Mureș, Gheorghe Marinescu Street No. 38, 540136 Târgu Mureș, Romania; oanam93@yahoo.com; 2 Department of Pediatrics 1, University of Medicine, Pharmacy, Sciences and Technology George Emil Palade from Târgu Mureș, Gheorghe Marinescu Street No. 38, 540136 Târgu Mureș, Romania; 3 Clinics of Pediatrics, Emergency County Clinical Hospital, Gheorghe Marinescu Street No. 50, 540136 Târgu Mureș, Romania; kolleranamaria@gmail.com

**Keywords:** *Helicobacter pylori* infection, immune thrombocytopenic purpura, children

## Abstract

In adults with immune thrombocytopenic purpura (ITP), the identification of *H. pylori* infection and its subsequent eradication proved to aid platelet recovery. Similar findings, at a smaller scale, were allegedly reported by some pediatric studies. This review’s objective was to establish the influence of *H. pylori* infection and its eradication upon platelet count and recovery in pediatric ITP. Three databases, namely Pubmed, Scopus and Web of Science, were searched for pediatric studies which investigated a link between *H. pylori* infection and thrombocytopenia. The search results retrieved a number of 21 articles which complied to the inclusion and exclusion criteria. Some studies report lower platelet values among children with ITP and documented *H. pylori* infection, as well as an improve in platelet numbers after *H. pylori* treatment. However, results are controversial, as multiple authors failed to identify a higher prevalence of *H. pylori* among children with ITP or a lack of significant change in therapeutic outcome with the addition of an eradication regimen to standard treatment. The main limitations of current pediatric studies remain the small study samples and the short follow-up periods of the included subjects. Hence, the long-term impact of *H. pylori* in children with ITP is still uncertain.

## 1. Introduction

Immune thrombocytopenic purpura (ITP) is defined as an isolated, transitory or persistent decrease in platelet count, under a diagnostic threshold of 100 × 10^9^/L, mediated by an immune process. The term, “immune”, is nowadays preferred to the old “idiopathic” label, in order to highlight a disorder of the immune system characterized by the coating of platelets with autoantibodies that act against platelet membrane antigens and inhibit megakaryocyte function [1,2]. Immunoglobulin G (IgG) antibodies bind to platelet membrane glycoproteins, which leads to the sequestration and phagocytosis of platelets within the spleen and can simultaneously impair platelet production [3,4]. The absence of a known cause or other underlying disorders responsible for the autoimmune thrombocytopenia suggests a diagnosis of primary ITP, whereas secondary ITP refers to each type of immune-mediated thrombocytopenia with the exception of primary ITP [2].

The result is a decrease in platelet numbers, which, depending on its severity, can lead to cutaneous hemorrhages such as petechiae and ecchymoses, mucosal bleedings including epistaxis or genito-urinary hemorrhages [5]. Severe bleeding complications, such as intracranial hemorrhage, occur with a general frequency of 1/800 at pediatric ages, in both newly onset and chronic ITP forms and are the main cause of long-term morbidity and mortality [5,6]. In spite of the potential severe outcome of ITP, which prompts for early recognition of the disease, its diagnosis still remains a challenge, as it involves exclusion of other causes of isolated thrombocytopenia [7].

In children, acute ITP prevails, usually develops a few weeks after a viral infection and has a self-limiting evolution in most cases, while chronic ITP develops in approximately 20% of the cases, especially in teenagers [8,9,10,11]. As pediatric ITP rarely becomes chronic, the incidence of ITP in children is estimated at 5 cases/100,000 children/year, significantly lower than the one in adults [12,13]. Recent data sustain the definition of chronic ITP as thrombocytopenia (<100,000/µL) which lasts for more than 12 months, whereas a 3–12-month interval of thrombocytopenia qualifies as persistent thrombocytopenia [2]. Risk factors for ITP chronicity include age at diagnosis, female sex and platelet count at the time of the diagnosis [14].

Primary ITP accounts for 80–90% of adult cases [3,15]. In similar fashion, around 20% of children cases can be attributed to secondary causes, according to a Spanish study [16]. *H. pylori* has been regarded as one of the potential causes of secondary ITP, but it is yet unclear whether treatment of this particular infection might be sufficient to improve platelet numbers [17,18]. There are several hypotheses that link *H. pylori* to ITP. Firstly, molecular mimicry plays a huge part. This is characterized by specific bacterial antibody production that cross-react with platelet glycoprotein antigens [19]. A specific cross-reactivity between platelet antigens and the cytotoxin-associated gene A (CagA) has also been described [20], together with a higher prevalence of CagA *H. pylori* positive strains among populations diagnosed with ITP [21]. Lewis (Le) antigens, expressed by particular *H. pylori* strains, adhere to platelets, which are consequently targeted by anti-Le antibodies in genetically susceptible patients. These also interfere with ITP pathogenesis through the same molecular mimicry-related processes [22]. Particular enhancement of platelet phagocytosis by the monocytes in *H. pylori* infected individuals seems to be involved in the development of secondary ITP as well [23]. Particular haplotypes such as HLA-DRB1*11,*14 and -DQB1*03 are more frequently encountered in ITP patients infected with *H. pylori*, whereas HLA -DRB1*03 is more rarely found within the same individuals [24]. Moreover, intestinal microbiota dysbiosis, which appears in the setting of *H. pylori* infection, has also been linked to ITP pathogenesis [25]. However, after *H. pylori* eradication, autoantibodies disappear after a period of one to two years, whereas a positive platelet response is seen in some studies after only one to two weeks, which suggests that other intricated mechanisms might play a role in the pathogenesis of ITP as well [26]. 

An association between *H. pylori* infection and ITP was proven for the first time in 1998, by Gasbarrini et al. [27]. Since then, the beneficial effect of *H. pylori* eradication over platelet numbers has been proved in adult patients with moderate ITP forms in studies with a randomized controlled trial (RCT) design [28]. In children, evidence promoting *H. pylori* eradication in ITP patients exists, but the sole influence of *H. pylori* in pediatric ITP is put into question [29,30]. Starting from various hypotheses that sustain the benefit of *H. pylori* treatment in improving platelet numbers in patients with ITP, this comprehensive review aims to establish the influence of *H. pylori* infection and eradication upon platelet counts and recovery in pediatric ITP.

## 2. Materials and Methods

We searched the Pubmed, Scopus and Web of Science databases for all articles indexed through the 25 May 2023, which assessed the relationship between *H. pylori* and thrombocytopenia in children. The search terms used were “Helicobacter” OR “*Helicobacter pylori*” AND “thrombocytopenia” AND “child”. We only aimed to include population-based studies (including prospective cohort and observational studies, retrospective observational studies, longitudinal studies and RCTs) conducted on child populations or studies enrolling both adults and children which conducted a separate analysis on the impact of *H. pylori* infection and/or its eradication on thrombocytopenia in pediatric populations. Exclusion criteria consisted of non-English language literature data, meta-analyses, review articles, case reports/series, editorials, letters to editor, as well as experimental, animal studies and adult population-based studies. Reference lists of the selected articles were also screened for compliance with the inclusion and exclusion criteria, to the review’s objectives and for database indexing.

The article selection process firstly consisted of exclusion of duplicates and triplicates, a task which was performed by authors SM and KAM. Each of the three authors of the article examined the title and abstracts of the identified reports in order to exclude irrelevant articles for the reviewer’s objectives. SM and KAM accessed the full-length text of the elected manuscripts and checked for compliance to the aforementioned inclusion criteria. Eventual disagreements between authors had been thoroughly debated and discussed by all authors. The inclusion of each individual record belonging to this review was established upon mutual agreement. 

The following information were extracted from articles belonging to the final selection pool: author name, year of publication, type of the study, target population, study group division and main findings of the article related to *H. pylori* prevalence among children with ITP, the impact of its presence on platelet count and the effect of its eradication upon platelet recovery rates. 

## 3. Results

**Figure 1:**
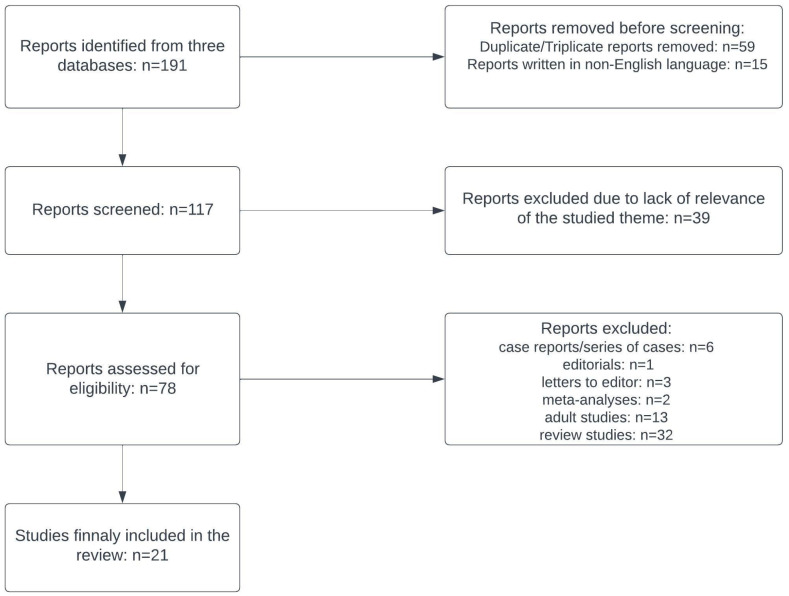
PRISMA flowchart with the eligible studies.

The initial research resulted in a total of 191 records. After exclusion of 59 duplicate and triplicate articles, 15 non-English language articles and 39 articles which were not in line with the reviewer’s objectives, a number of 78 relevant articles were screened. Review articles, meta-analyses, case reports/series, editorials, letters to editor (not reporting results of original studies) were excluded, together with studies conducted on adults. This systematic selection resulted in 21 admissible articles. The article selection process has been detailed in Figure 1, being performed in accordance with the PRISMA 2020 statement [31]. 

A summary of the main findings of the pediatric studies which complied to our election criteria has been provided through Table 1.

Although the causative role of *H. pylori* in pediatric ITP has not been sufficiently investigated, some studies sustain a test-and-treat strategy in children diagnosed with this particular condition. Russo et al. enrolled an impressive pediatric cohort of 244 patients diagnosed with chronic ITP and proved that platelet recovery rates were significantly higher in those patients in whom *H. pylori* was successfully eradicated, when compared to spontaneous remission rates encountered in *H. pylori* negative patients [29]. Brito et al. conducted an RCT on 85 children, including 22 children and adolescents with chronic ITP, equally randomized into a treatment group and a control group with similar baseline platelet counts. The authors found no significant difference in complete platelet response rates, defined as platelet values exceeding 150 × 10^9^/L, between treated and untreated children. However, similarly to the study of Russo et al. [29], *H. pylori* eradication yielded a more frequent complete platelet response than in the case of uninfected patients [32]. In accordance with these findings, the study of Ferrara et al. sustains *H. pylori* screening in chronic, childhood and adolescence ITP, after showing a more frequent complete platelet response in patients in whom *H. pylori* was successfully treated. No differences were seen in this study in pre-therapeutic platelet counts in relation to *H. pylori* presence [33]. Another Ethiopian study found a significant reduction in platelet numbers and mean platelet volume in children with positive *H. pylori* stool antigen test or positive *H. pylori* antibodies [34]. 

The beneficial effects of *H. pylori* eradication in children with ITP are still being investigated. The numerically limited cohorts of other studies greatly influence the interpretation of their results. A Dutch study sustained the favorable therapeutic response brought up by *H. pylori* eradication in chronic ITP, but its conclusion cannot be perceived at a larger scale, as only 3 children out of the entire study cohort were diagnosed with the infection [35]. In similar fashion, a small scale study conducted in Iran found that *H. pylori* eradication might facilitate a sustained therapeutic response in chronic pediatric ITP, but this infection was diagnosed in a very small subset of patients, as well [36]. Jaing et al. reported a sustained rise in platelet counts after 6 months of follow-up in five out of nine children infected with *H. pylori*, but due to the limited number of patients included in the study, it is unclear whether infection eradication, the course of the disease or standard treatment played the major role in therapeutic favorable outcome [37]. Thus, it is hard to interpretate the impact of *H. pylori* eradication in very small subsets of patients with chronic ITP, which characterize most pediatric studies. For example, Hayashi et al. identified *H. pylori* in only two out of ten patients with chronic ITP and reported a sustained response after 1 year of follow up in only one of these [38]. A larger subgroup, of 280 patients under the age of 18 years, was though included in a nationwide study, conducted in Taiwan. Within this study, *H. pylori* was only associated with adult ITP, the infection being absent in each of the children included [39]. 

Prevalence of *H. pylori* infection varies with age and geographical region among subjects with ITP [52]. In an adult study enrolling Italian and British populations, the incidence of *H. pylori* infection in patients with ITP was reported to be similar to the one of the general, healthy population [53]. However, one study examining spleen specimens from patients undergoing splenectomy for ITP or trauma identified a higher prevalence of *H. pylori* in the first group. Moreover, the presence of *H. pylori* infection was associated with low expression of the FC gamma receptors IIB (FCGRIIB) within splenic macrophages, which is known to play a role in the etiology of ITP [54]. Furthermore, within a case–control study, Abdollahi et al. reported a higher prevalence of *H. pylori* infection among a pediatric group with ITP, when compared to healthy controls. The study did not analyze the therapeutic outcome in the case group, nor the impact of *H. pylori* eradication [40]. On the other hand, a Finnish study conducted on 17 children with ITP failed to confirm the presence of *H. pylori* infection in any of these subjects [41]. Similarly, one study conducted in Japan which analyzed remission rates of chronic ITP in children, during a follow-up period of 20 years, did not identify the presence of *H. pylori* in any of the included subjects [42]. A slightly higher prevalence of *H. pylori* than in general in population was found in a Taiwanese case–control study, but this difference did not reach statistical significance. The relationship between *H. pylori* infection and platelet response was also analyzed, revealing that its presence is slightly discernible in the course of chronic ITP [43]. 

The sole presence of *H. pylori* infection might not additionally impact platelet counts and clinical picture in pediatric ITP. Similar platelet values between children study groups were reported, divided based on *H. pylori* infectious status. One study compared multiple hematological parameters between children diagnosed with *H. pylori* gastritis, those diagnosed with non-*H. pylori* gastritis and a control group with functional gastro-intestinal symptoms, without microscopic anomalies of the gastric mucosa. Platelet numbers were found to be similar to the ones of healthy subjects in both study groups [44]. Moreover, the study of Afifi et al. also compared various erythrocyte parameters and platelet numbers between pediatric subjects with *H. pylori* infection and healthy controls. Significant lower values of the mean corpuscular volume (MCV) and mean cell hemoglobin (MCH) were found in the *H. pylori* positive group, but platelet counts did not present significant variations among the two study groups [45]. 

Hence, uncertainty still surrounds the theory of *H. pylori*’s implications in the pathogenesis of ITP, as Bisogno et al. proved within a small-scale study. The number of patients in whom the bacterial infection was not confirmed and presented spontaneous improvements of platelet counts was similar to the one of patients in whom bacterial eradication showed a beneficial, yet unsustainable, therapeutic response [46]. Efforts to eradicate *H. pylori* might not produce the expected positive outcome over platelet numbers. A multi-center randomized controlled trial showed that *H. pylori* eradication does not bring any improvement in platelet counts, after random assignment of 16 children into two balanced groups of patients, one who benefited from eradication therapy and the other one in which anti-infectious treatment was not considered. An important number of subjects from this particular study presented cytotoxin-associated gene A (CagA) and/or vacuolating cytotoxin A (VacA) antibodies [51]. Loffredo et al. also reported no difference in therapeutic outcome from patients without *H. pylori*, after single or multiple courses of eradication lines were applied to children in whom bacteria was positive upon detection of at least two non-invasive tests [47]. Another study confirmed that *H. pylori* eradication does not bring any additional benefit to standard treatment in children with chronic ITP [48]. *H. pylori* infection does not seem to interfere with short term response to intravenous immunoglobulin (IvIg) treatment, assessed after a time-span of 2 weeks, according to Morimoto et al. [49]. Furthermore, a case–control study described no significant differences in number of subjects infected with *H. pylori*, when comparing children diagnosed with ITP and healthy controls. Within the study group, the therapeutic outcome was also assessed in relation to *H. pylori* infectious status and the bacterial pathogen was found to have no impact on platelet count response [50]. Therefore, it is still unclear if *H. pylori* eradication improves therapeutic outcome in pediatric ITP.

## 4. Discussion

Extra-gastric manifestations of *H. pylori* infection seem to be largely represented by hematological disorders, such as iron deficiency anemia (IDA), megaloblastic anemia (caused by vitamin B12 deficiency) and ITP [55,56,57]. This review has focused on the impact that *H. pylori* infection poses upon the clinical course and therapeutic efficacy in pediatric ITP, starting from the long-running debate and controversy surrounding the importance of *H. pylori* eradication in patients with ITP. An update to the pediatric literature data has been provided through inclusion of more recent studies conducted on the subject, which met the inclusion and exclusion criteria, but which are still hindered by several limitations. An older review article, published in 2005, initially pointed out that studies analyzing a relationship between *H. pylori* and ITP which were conducted to that point were small-scale, and the enrolled populations were not followed up for longer periods of time [58]. The studies included in this review are also characterized by numeric limitation of target groups. Moreover, only two of those were longitudinally designed, but the follow-up period was limited to 6 months and one year, respectively. 

With the availability of new data derived from adult studies which showed utility of *H. pylori* treatment, in cases where the bacterium was encountered, the 2011 guidelines of the American Society of Hematology recommended against routine screening of *H. pylori* infection in patients with ITP, but supported treatment of the bacterial infection in those patients in whom non-invasive or invasive diagnostic tests delivered positive results [6]. Afterwards, the 2019 guidelines of the American Society of Hematology maintained the same position, recommending against routine testing for *H. pylori*, which was meant to be limited to those subjects with clinical, suggestive symptoms [59]. On the other hand, the 2010 Consensus Guidelines of the Associazione Italiana di Ematologia e Oncologia Pediatrica (AIEOP) recommended the conductance of *H. pylori* stool antigen tests in children with ITP, in order to search for an underlying infectious cause which can be combated [60]. As listed in Table 1, in most of the studies included in this review, the identification of *H. pylori* infection was subject to stool antigen testing as well. However, a handful of these studies assessed the presence of the infection only in a subset of patients. Moreover, only a third of the studies included in this review evaluated platelet outcome after eradication therapy was instituted, with the other studies only assessing response to standard ITP treatment in relation to the presence/absence of *H. pylori* infection. Still, the presence of *H. pylori* in subjects with ITP might prompt the physician towards recommending a therapeutic scheme. One treatment strategy protocol developed based on an adult study conducted in a referral center from Northern Brazil also proposes a screening of *H. pylori* and its subsequent treatment, if diagnostic test results are positive [61]. 

The success of *H. pylori* eradication in patients with ITP varies greatly among different studies. Eradication rates lower than 1% or 5% have been reported, or even exceeding 60% in research conducted in Italy and Japan [27,62,63]. Stasi et al. found among an adult cohort diagnosed with ITP a similar prevalence of *H. pylori* infection to the one found in general population. Furthermore, the authors described a beneficial effect of *H. pylori* eradication only in those mild to moderate cases of ITP, with recent onset [53]. A French study also reported a similar seroprevalence of *H. pylori* infection between a study group of adults with ITP and controls, whereas a Columbian study identified a compellingly higher prevalence of the same bacteria within an ITP cohort, when compared to healthy subjects [64,65]. Still, *H. pylori* seems to be encountered more rarely in children than in adults suffering from ITP [52]. Kuwana et al. have reviewed the heterogeneity of *H. pylori* therapeutic response rates in subjects with ITP, reporting higher eradication failure rates in the United States and non-Italian European countries. Furthermore, the authors claimed that the very low incidence of *H. pylori* infection reported within pediatric studies questions the utility of the specific eradication treatment among these patients, as opposed to adult counterparts [66]. As a matter of fact, from the reports included in this review, one study conducted in Taiwan and another one enrolling a Finish pediatric population failed to identify *H. pylori* in any of the enrolled subjects [39,41].

Secondary ITP forms are accompanied by a more pronounced thrombocytopenia, splenomegaly, hepatomegaly and lower hemoglobin counts, according to an adult study [67]. In children in particular, search for a secondary cause of ITP and treatment of possible causative agents could represent an important therapeutic aid which can lead to avoidance of standard treatment and its widely known side effects, which are more extensive at this particular age group [68]. One study conducted on a numerous cohort of pediatric ITP patients, identified that the two most frequent causes of secondary ITP cases are recent immunizations and viral infections, followed by autoimmune disorders and immunodeficiency [16]. Within this study, post-immunization and post-viral infections ITPs showed the best remission rates [16]. Although *H. pylori* infection is usually acquired during childhood, physicians are less familiar with its extra-gastric, hematological manifestations. Hence, screening of this bacterium is not routinely performed in children with ITP and is reported as a rare practice among adult studies as well [69,70]. One meta-analysis of six randomized trials sustained the importance of *H. pylori* eradication in adults with ITP, but failed to identify a therapeutic benefit in children diagnosed with the same condition, in whom the infection was successfully treated [71]. One systematic review study drew this same conclusion, in light of the frequent limitations of pediatric studies addressing a potential association between *H. pylori* and ITP, such as the lack of controls, the small sample sizes, the low prevalence of *H. pylori* infection among the studied subjects and the short period of follow-ups. Furthermore, the same review concluded that the poor statistical power of pediatric studies hinders the establishment of benefits to *H. pylori* eradication in children, in both gastric and extra-gastric manifestations [72]. Another meta-analysis of 7 Middle-Eastern, adult studies, highlighted the same advantages of *H. pylori* screening and treatment in ITP, but was hindered by several limitations, which the authors acknowledged. Among these, the small population samples, the heterogeneity of the *H. pylori* detection methods used and of the complete versus partial platelet response criteria were the ones that stood out [73]. Although a correlation between *H. pylori* eradication and platelet count increase in children seems obvious, another meta-analysis of miscellaneous studies points out their limitations related to the design, and the need for further evidence delivered by RCTs [74]. Unicentric studies, even those performed on significantly larger cohorts, identified *H. pylori* only in small subsets of patients with ITP and found very low platelet recovery rates in case of successful infection eradication [75]. Hence, due to the bias-prone studies conducted in both pediatric and adult populations, the utility of a test and treat strategy for *H. pylori* is still in question. Moreover, as shown in Table 1, most of the studies included in this review, which assessed the effect of *H. pylori* eradication, showed no significant improvement in platelet counts.

As the persistence of chronic gastric inflammation might be responsible for mild thrombocytosis, controversial results were reported by Matsukawa et al., who claimed through their study that *H. pylori* eradication leads to a decrease in platelet numbers in adults with gastritis and gastric ulcer, as opposed to non-eradicated patients [76]. However, one study, which assessed the impact of chronic gastric inflammation upon platelet numbers and mean platelet volume (MPV), found no significant differences between two study groups of subjects, divided depending on *H. pylori* infectious status. Furthermore, MPV did not correlate with severity of chronic gastric inflammation [77]. Two pediatric studies also confirmed these findings, describing lack of significant differences in MPV values between non-*H. pylori* gastritis and *H. pylori* gastritis subjects, eradicated and non-eradicated patients, and lack of correlations between this parameters and different degree of gastritis severity [44,78]. Mean platelet volume has been for a long time regarded as a marker of platelet activation, which might increase with enhanced thromobocytopoiesis in inflammation and infections [79]. Therefore, it is yet unclear whether concurring *H. pylori* infection and chronic gastritis influence platelet parameters in both adults and children.

In various adult cohorts, the eradication of *H. pylori* has been followed by a sustained, favorable platelet response after follow-up periods of one year [80]. However, platelet response rates seem to be higher in patients with milder thrombocytopenia and countries with higher prevalence of *H. pylori* infection [81]. One previous systematic review of studies performed on pediatric populations concluded that platelet response rates in pediatric populations are expected to be similar to the ones found so far in adults and that *H. pylori* eradication should constitute a first-line treatment approach in children with ITP as well [82]. There is still a discrepancy between the results reported by cohort studies, such as those included in the review, and individual case reports which sustain the need for *H. pylori* infection eradication in pediatric ITP. The case of a 12-year-old male patient who presented recovery of platelet counts after *H. pylori* eradication distinguishes among others, due to a reported higher efficacy of the bacterial eradication when compared to corticosteroid treatment [83]. In another case report on an 11-year-old, a sustained platelet response was reported only after *H. pylori* re-eradication, in spite of previous therapy with IvIg and cepharantine, administered as a substitute of corticosteroids [84]. The report of a female of 13 years of age with *H. pylori* induced atrophic gastritis also details the remission of the associated conditions, namely IDA and ITP, with infection eradication [85]. A temporary increase in platelet numbers was also reported in relation to omeprazole monotherapy [84], which had previously been described to eventually lead to *H. pylori* eradication and to remission of ITP, when administered for a period of approximately 1 year and a half in an elderly woman [86]. Ikeda et al. describe a peculiar case of neonatal thrombocytopenia related to maternal ITP, diagnosed during pregnancy. The neonate benefited from IvIg treatment, but in the mother’s case, ITP was found to be related to *H. pylori* and infection eradication led to recovery of platelet counts. Moreover, the case brought into attention the neonatal impact of gestational platelet auto-antibodies [87]. Although case reports offer insufficient evidence to sustain *H. pylori* screening and treatment in pediatric ITP, they prove that for certain individuals, this strategy might be helpful in improving platelet numbers. 

In spite of the multiple pediatric studies which have thoroughly investigated the impact of *H. pylori* infection and its eradication upon platelet counts, their main limitations remain the small population samples of available studies and the short follow-up periods of the included patients, which have been highlighted through Table 1. Furthermore, there are currently only three RCTs [32,48,51] which randomized children with ITP into two groups, depending on the choice of adding *H. pylori* treatment to conventional therapy. Hence, available pediatric studies with higher statistical power, such as RCTs, are very scarce. Therefore, there are insufficient data available to certify the negative impact of *H. pylori* infection upon platelet count in pediatric populations [88], and the long-term stability of platelet numbers after *H. pylori* eradication is yet uncertain in children.

## 5. Conclusions

The generally low prevalence of *H. pylori* in children with ITP might suggest that this infection only plays a minor role in the pathogenesis of this condition, at pediatric ages. Controversy still surrounds the utility of *H. pylori* identification and treatment in children with ITP. Several studies reported the benefits of *H. pylori* screening, but evidence is scarcer than in adult populations and it is yet unclear how the treatment of this particular infection influences platelet counts in the long term. The lack of longitudinal design of most of the available pediatric research calls for future studies which should also consistently follow up the enrolled study populations.

## Figures and Tables

**Table 1:** Characteristics of pediatric studies which assessed the impact of *H. pylori* infection and eradication upon the course of platelet counts in patients with ITP.

Reference (Author, Year)	Type of Study	Study Group Division	*H. pylori* Detection Method	Main Outcome
Russo et al., 2011 [29]	Prospective, case–control multicentric study	244 children with chronic ITP who were screened for *H. pylori* infection: 50 children positive for *H. pylori* (37 children receiving eradication therapy) 194 children negative for *H. pylori*	Stool antigen testing in most cases Urea breath test conducted in 2 cases	Significantly higher platelet response rates among patients who underwent successful eradication in comparison with spontaneous remission rates among *H. pylori* negative patients
Brito et al., 2015 [32]	RCT	85 children with chronic ITP: 63 patients negative for *H. pylori* 22 patients positive for *H. pylori:* 11 children randomized in the treatment group 11 children randomized in the control group	Stool antigen test + urea breath test	Significantly higher complete platelet response rates among children who received specific *H. pylori* eradication therapy
Ferrara et al., 2009 [33]	Retrospective observational study	24 children with chronic ITP: 8 children positive for *H. pylori* 16 children negative for *H. pylori*	Stool antigen test	Significant increase in platelet counts after eradication therapy among *H. pylori* infected children; no significantly difference in platelet numbers during the follow-up period among the uninfected patients
Baxendell et al., 2019 [34]	Cross-sectional study	1038 school children: 343 children infected with *H. pylori* 695 children negative for *H. pylori*	Stool antigen test + *H. pylori* antibody test	Significantly lower platelet and MPV values in children infected with *H. pylori* in comparison with uninfected counterparts
Neefjes et al., 2007 [35]	Observational study	47 children: 3 children infected with *H. pylori* 44 children negative for *H. pylori*	Stool antigen test	Significant platelet response within the children who benefited from eradication therapy
Maghbool et al., 2009 [36]	Retrospective study	30 children: 5 children infected with *H. pylori* 25 children negative for *H. pylori*	Stool antigen test	Platelet response was consistent after 1 year of follow-up in 4 of the *H. pylori* infected patients after eradication
Jaing et al., 2003 [37]	Observational study	22 children: 9 children infected with *H. pylori* 13 children negative for *H. pylori*	Stool antigen test	5 patients from the 9 infected with *H. pylori* showed partial or complete remission of platelet counts after 6 months, this response being sustained for a medium period of 16 months
Hayashi et al., 2005 [38]	Observational study	10 children: 2 children infected with *H. pylori* 8 children negative for *H. pylori*	Stool antigen test + urea breath test	A sustained platelet response was reported in only one of the *H. pylori* patient during a follow-up period of over an year
Wu et al., 2018 [39]	Case-control study	280 children with chronic ITP	Stool antigen test	*H. pylori* infection was not identified in any of the children with ITP
Abdollahi et al., 2015 [40]	Case-control study	106 children: 42 children with ITP 64 healthy controls	Stool antigen test	Significantly higher prevalence of *H. pylori* infection among the case group of children with ITP
Rajantie et al., 2003 [41]	Observational study	17 children with chronic ITP	Stool antigen test + urea breath test + *H. pylori* IgA/IgG antibodies	*H. pylori* infection was absent in each of the children included in the study
Kim et al., 2016 [42]	Retrospective observational study	200 children with ITP	Stool antigen test	Absence of *H. pylori* infection among 5 children with chronic ITP whose stool antigen test were examined
Wu et al., 2007 [43]	Case-control study	62 children: 32 children with ITP 30 children in the control group	Stool antigen test	No significant differences between the two study groups in terms of *H. pylori* prevalence rate Similar characteristics and treatment response ratio between *H. pylori* positive and *H. pylori* negative subjects from study group
Săsăran et al., 2020 [44]	Prospective, case–control study	151 children: 31 patients with *H. pylori* gastritis 53 patients with non- *H. pylori* gastritis 67 healthy controls	Histology based diagnosis	No significant differences in terms of platelet number and MPV values between the three study groups Platelet numbers and MPV values did not vary with different degrees of gastritis
Afifi et al., 2011 [45]	Case-control study	120 children: 60 patients with *H. pylori* positive antibodies 60 controls with *H. pylori* negative antibodies	*H. pylori* antibody testing	No significant differences in platelet counts within the two study groups
Bisogno et al., 2008 [46]	Longitudinal study	24 children with ITP: 8 children infected with *H. pylori* 16 children negative for *H. pylori*	Stool antigen test + urea breath test	No significant differences in platelet counts between the two study groups Improvement of platelet count in 3 patients who underwent eradication therapy, after six months
Loffredo et al., 2007 [47]	Longitudinal study	39 children with ITP: 8 children infected with *H. pylori* 31 children negative for *H. pylori*	Stool antigen test + urea breath test + *H. pylori* specific serum IgG antibodies	No improvement in platelet counts in children in whom *H. pylori* was successfully eradicated No significant differences in platelet counts between infected and uninfected patients during a follow-up period of one year
Eghbali et al., 2019 [48]	Open label RCT	28 children with ITP and *H. pylori* infection: 14 children receiving ITP and *H. pylori* therapy 14 children receiving ITP therapy	Stool antigen test	No significant differences between the two study groups in terms of platelet values at the baseline and after six months of follow-up
Morimoto et al., 2014 [49]	Retrospective observational study	49 children with primary ITP (18 children in whom *H. pylori* infectious status was examined): 7 children positive for *H. pylori* infection 11 children negative for *H. pylori* infection	Stool antigen test/blood antigen test/urine antibody test	Short-term response to intravenous Ig treatment was not affected of *H. pylori* infection
Jaing et al., 2006 [50]	Case-control study	91 children: 63 patients with acute ITP 28 healthy controls	Stool antigen test	Similar prevalence of *H. pylori* infection between the two study groups Similar response to steroid treatment among the study group, irrespective of *H. pylori* infectious status
Treepongkaruna et al., 2009 [51]	Multicenter RCT	55 children with chronic ITP: 16 patients with *H. pylori* infection who were randomized as follows: ○ 7 children in the treatment group ○ 9 children in the control group	Stool antigen test	*H. pylori* eradication did not bring any improvement in platelet count CagA antibodies were positive in 12 patients with documented *H. Pylori* infection, whereas VacA antibodies were positive in 7 patients

Legend: CagA—cytotoxin-associated gene A; *H. pylori*—*Helicobacter pylori*; Ig—immunoglobulin; ITP—immune thrombocytopenic purpura; MPV—mean platelet volume; RCT—randomized controlled trial; VacA—vacuolating cytotoxin A.
